# PUM1 knockdown prevents tumor progression by activating the PERK/eIF2/ATF4 signaling pathway in pancreatic adenocarcinoma cells

**DOI:** 10.1038/s41419-019-1839-z

**Published:** 2019-08-08

**Authors:** Haisu Dai, Kaicheng Shen, Yishi Yang, Xingxing Su, Yuandeng Luo, Yan Jiang, Ling Shuai, Ping Zheng, Zhiyu Chen, Ping Bie

**Affiliations:** Department of Hepatobiliary Surgery, Southwest Hospital, Third Military Medical University (Army Medical University), 400038 Chongqing, China

**Keywords:** Targeted therapies, Oncogenesis

## Abstract

Pancreatic ductal adenocarcinoma (PDAC) is a malignant tumor with very poor prognosis. Therefore, it is important to fully understand the molecular mechanism underlying its occurrence and development. Pumilio RNA-binding family member 1 (PUM1) has been reported to function as an oncogene in ovarian cancer and nonsmall cell lung cancer. However, its role and mechanism in PDAC have not been fully illuminated. Here, we found that the PUM1 protein levels were higher in PDAC tissues than in adjacent tissues and that PUM1 levels were significantly associated with TNM stage and overall survival time, indicating a correlation between high PUM1 expression and poor prognosis in patients with PDAC. In vitro and in vivo assays showed that PUM1 knockdown inhibited cell proliferation, migration, invasion, and epithelial–mesenchymal transition (EMT), and promoted apoptosis in MIA PaCa-2 and PANC-1 cells. Through cDNA microarrays and ingenuity pathway analysis, we found that the activation of the eIF2 signaling pathway significantly correlated with PUM1 knockdown. These results were further confirmed by the increased levels of key components of the eIF2 signaling pathway, p-PERK, p-EIF2A, and ATF4 in PUM1 knockdown cells. We also found that PUM1 levels have a significant negative correlation with p-PERK levels in PDAC tissues and that PERK overexpression inhibited cell proliferation, migration, invasion, and EMT, and promoted apoptosis in vitro. Moreover, a PERK inhibitor alleviated the effects of PUM1 knockdown on cell proliferation, apoptosis, migration, invasion, and EMT. Taken together, our results revealed that PUM1 knockdown suppressed cell growth, invasion, and metastasis, and promoted apoptosis by activating the PERK/eIF2/ATF4 signaling pathway in PDAC cells. PUM1 could be a potential target to develop pharmaceuticals and novel therapeutic strategies for the treatment of PDAC.

## Introduction

Pancreatic cancer is extremely malignant and ranks as the seventh leading cause of cancer-related death and the second leading cause of digestive tract malignancy-related death^1^. Surgical treatment shows no satisfactory effects, and the 5-year postoperative survival rate is below 5%^2^. Therefore, it is important to further understand the molecular mechanism underlying the occurrence and development of pancreatic cancer, as this will provide a theoretical basis to find new targets and develop therapies.

Pumilio RNA-Binding Family Member 1 (PUM1) is a member of the PUF family, that shows high homology with the Pumilio proteins of *Drosophila* and the fem-3 mRNA-binding factors of *Caenorhabditis elegans*^3^. PUM1 can suppress the translation of target mRNAs by binding to their 3ʹ untranslated region^4,5^. It has been reported that PUM1 is upregulated and functions as an oncogene by promoting cell proliferation, migration, and invasion in ovarian cancer^6^. MicroRNA-411–5p acts as a tumor suppressor by inhibiting PUM1 mRNA translation in non-small cell lung cancer (NSCLC), which indicates that PUM1 also functions as an oncogene in this disease^7^. However, there is no information regarding the function of PUM1 in pancreatic cancer, and the role of PUM1 in regulating carcinogenesis has not been fully elucidated. Therefore, it is necessary to further discuss the role of PUM1 in the development of pancreatic cancer.

Here, we aimed to evaluate the relationship between PUM1 protein levels and clinical characteristics of pancreatic ductal adenocarcinoma (PDAC) patients, the most common form of pancreatic cancer, accounting for ~85% of the cases. In addition, we evaluated the function of PUM1 on apoptotic, proliferative, invasive, and metastatic behaviors in PDAC cells through in vitro and in vivo experiments. Moreover, we explored the association between the eIF2 signaling pathway and PUM1 through cDNA microarrays and Ingenuity Pathway Analysis (IPA). Finally, we analyzed the relationship between PUM1 and the PERK/eIF2 signaling pathway, a regulator of the endoplasmic reticulum stress (ERS) response^8^, to further explore the regulatory mechanism of PUM1 in PDAC.

## Materials and methods

### The Cancer Genome Atlas (TCGA) and Gene Expression Profiling Interactive Analysis (GEPIA) database analysis

The expression profiles of PUM1 mRNA in pancreatic adenocarcinoma (PAAD) tissues were analyzed using the GEPIA database^9^. The association between the PUM1 mRNA expression level and the overall survival time of patients with PAAD was analyzed by referring to TCGA database^10^.

### Patient information and follow-up study

In this study, we enrolled 60 patients who were diagnosed with PDAC from 2010 to 2014 in the Southwest Hospital, Third Military Medical University. PDAC tissues were collected during surgery. None of the patients received chemotherapy before the surgery. After surgery, a one-year follow-up study was conducted to record overall survival. This study was approved by the research ethics committee of Southwest Hospital, Third Military Medical University. Written informed consent was obtained from all the patients before they were enrolled in the study.

### Tissue microarrays analysis

Tissue microarrays were prepared, and the expression level of PUM1 and p-PERK in PDAC tissues was examined by immunohistochemical analysis by Shanghai Outdo Biotech Co., Ltd. The obtained immunohistochemical results were reviewed independently by two senior pathologists according to a method described previously^11^.

### Cells and reagents

Human PDAC cell lines, BxPC-3, AsPC-1, MIA PaCa-2, and PANC-1, and human pancreatic duct epithelial cells HPDE6-C7 were purchased from the Cell Bank of the Chinese Academy of Sciences (Shanghai, China). The PUM1 primary antibody was purchased from ABclonal Technology (Wuhan, China). The remaining antibodies were provided by Abcam (Cambridge, MA, USA). The PERK inhibitor GSK2606414^12^, was purchased from Selleckchem (Houston, TX, USA).

### Construction of PUM1 stable knockdown cells, and cell treatment

The lentivirus expressing the shRNA of PUM1 (shPUM1) was provided by GenePharma (Shanghai, China). The targeting sequence of shPUM1 was AGCAACTAATTCAGCTAAT. To construct PUM1 stable knockdown cells, the lentivirus-expressing shPUM1 was used to infect MIA PaCa-2 and PANC-1 cells; empty lentivirus was used as the negative control (NC). To analyze the effect of the PERK inhibitor GSK2606414^12^ on the cells of the NC or shPUM1 group, the cells were treated with 0.05 μM GSK2606414 or DMSO at the same concentration.

### Construction and Transfection of PUM1 and PERK overexpression vectors

The full-length open reading frame of *PUM1* (NM_001020658) or *PERK* (NM_004836) were cloned and inserted into the expression vector pcDNA3.0. The resulting vectors are referred to as ov-PUM1 or ov-PERK. The empty vector pcDNA3.0 referred to as vector. The cells were transfected with the different vectors using Lipofectamine 2000 (Invitrogen, Carlsbad, CA, USA) according to the manufacturer’s instructions.

### RNA extraction and quantitative reverse transcription PCR (qRT-PCR)

Total RNA was extracted using TRIzol reagent (Invitrogen), and qRT-PCR was carried out according to a previously described method^13^. qRT-PCR reactions were performed in duplicate and repeated three times. Fold induction of gene expression was calculated using the 2^−ΔΔCT^ method.

### Western blotting

RIPA buffer was used to isolate the total protein of the cells. After quantitation of protein concentration, equal amounts of protein were separated using 10% SDS polyacrylamide gels, then transferred to PVDF membranes (Millipore, Billerica, MA, USA). After blocking for 1 h at 37 °C, the membranes were incubated with different primary antibodies at 37 °C for 1 h. After washing three times with TBS containing 0.05% Tween-20 (TBST), horseradish peroxidase (HRP)-conjugated secondary antibody was added, and incubation was carried out at 37 °C for 40 min; the membranes were then washed three times with TBST. An x-ray film was used for protein visualization using SuperSignal West Pico Plus (Thermo Fisher Scientific, Waltham, MA, USA). The relative protein expression level corresponds to the relative ratio of the integral optical density of the target protein to that of the reference protein GAPDH.

### Cell proliferation assay

The CellTiter 96 AQueous One Solution Cell Proliferation Assay kit (Promega, Madison, WI, USA) was used to examine cell proliferation after different treatments. The cell proliferation results were detected at 490 nm.

### Flow cytometry for analysis of cell apoptosis

PUM1 stable knockdown cells, NC cells, or cells treated with GSK2606414 or DMSO were obtained to determine the level of cell apoptosis using the Annexin V-FITC/PI apoptosis detection kit (Keygen, Nanjing, China) on the BD Accuri™ C6 flow cytometer (BD Biosciences, San Jose, CA, USA).

### Transwell assay

To detect the cellular migration ability, cells from all the groups described in this study were digested with trypsin and washed three times with serum-free medium. Approximately 10^4^ cells suspended in 100 µl serum-free medium were added to the upper chamber coated with Matrigel for the invasion assay and without Matrigel for migration assays, and incubated for 6 h. Culture medium (600 µl) containing 20% FBS was added to the lower chamber. After incubation at 37 °C in a humidified atmosphere with 5% CO_2_ for 24 h, cells in the upper surface were wiped off with cotton balls. Cells that migrated to or invaded the reverse side of the Transwell membrane were washed with PBS, fixed with 5% glutaraldehyde at 4 °C, and stained with crystal violet (0.5%) for 5–10 min. After washing with PBS twice, five microscopic fields were randomly observed to count the number of migratory or invasive cells.

### Subcutaneous xenograft tumor model

All animal experiments in this study were approved by the research ethics committee of Southwest Hospital, Third Military Medical University. After 3 days of adaptive feeding, athymic nude mice were divided into four groups (*n* = 8) and were subcutaneously injected in the right armpit region with PUM1 stable knockdown or NC MIA PaCa-2 and PANC-1 cells (4 × 10^6^ cells in 0.2 ml of PBS). Tumor size was measured using calipers 10, 13, 16, 19, 22, 25, and 28 days after injection, and tumor volume was calculated using the formula: (*L* × *W*^2^)/2 where (*L* is the length and *W* is the width of the tumor). Subcutaneous xenograft tumor tissues were isolated and fixed in 4% paraformaldehyde, and immunohistochemical analysis for PUM1 was performed.

### Lung metastasis mouse model

Four groups (*n* = 10) were injected intravenously in the tail with 5 × 10^5^ PUM1 stable knockdown or NC MIA PaCa-2 and PANC-1 cells resuspended in 100 μl of Hank’s Balanced Salt Solution. All mice were sacrificed 6 weeks after injection. Lung and liver tissues were isolated and fixed in 4% paraformaldehyde. Hematoxylin and eosin (H&E) staining and immunohistochemical analysis for Ki67 was performed.

### cDNA microarray and analysis

Total RNA was isolated from PUM1 stable knockdown or NC MIA PaCa-2 cells. cDNA microarrays were carried out on the GeneChip PrimeView Human Expression Array (Affymetrix, Santa Clara, CA, USA) by Shanghai Genechem Co., Ltd (Shanghai, China). mRNAs that were differentially expressed between the two groups were defined as those with a fold change ≥ 1.5 and *P* value < 0.05. IPAs^14^ were carried out by Shanghai Genechem Co., Ltd to identify the signaling pathways regulated by PUM1.

### Statistical analysis

Statistical analysis was performed using SPSS 19.0 software (IBM, Chicago, IL, USA). The results are shown as means ± standard deviations. Statistical comparison between the two groups was analyzed using Student’s *t* tests. Statistical analysis for more than two groups was carried out using one-way ANOVA followed by post hoc LSD test. The overall survival rate of patients with PDAC was evaluated based on PUM1 expression using Kaplan–Meier analysis. The correlation of PUM1 and p-PERK levels in PDAC tissues was analyzed by linear regression analysis using sing GraphPad Prism version 7.0 (GraphPad software, San Diego, CA, USA). A value of *P* < 0.05 was considered statistically significant.

## Results

### PUM1 expression is correlated with metastasis and prognosis

**Fig. 1:**
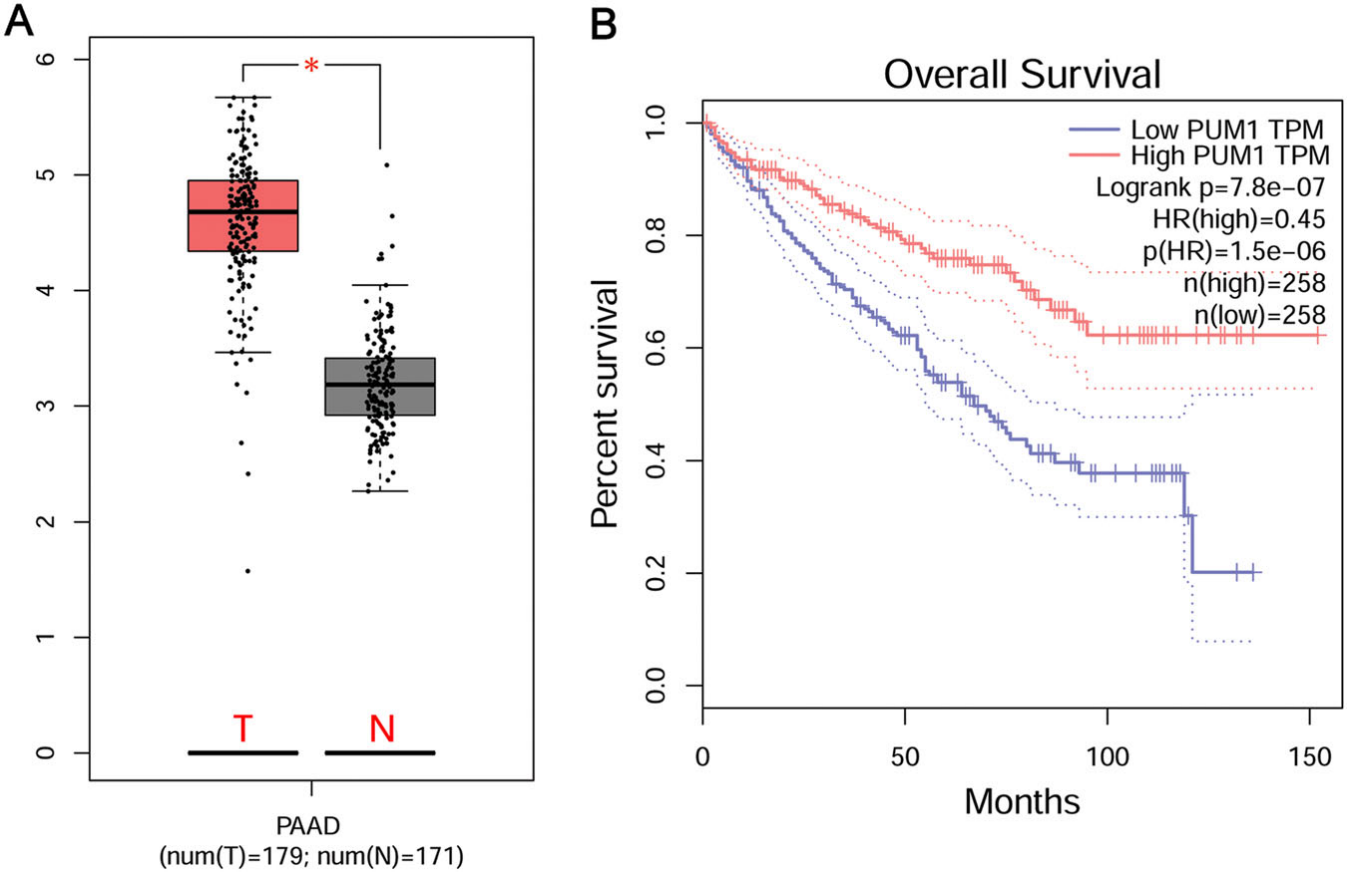
PUM1 mRNA expression is correlated with prognosis in pancreatic adenocarcinoma (PAAD). **a** PUM1 mRNA expression levels in PAAD tissues (T) and normal tissues (N) analyzed by gene expression profiling interactive analysis (GEPIA). *P < 0.05. **b** Analysis of the relationship between PUM1 mRNA expression and overall survival of PAAD patients analyzed using The Cancer Genome Atlas (TCGA)

**Fig. 2:**
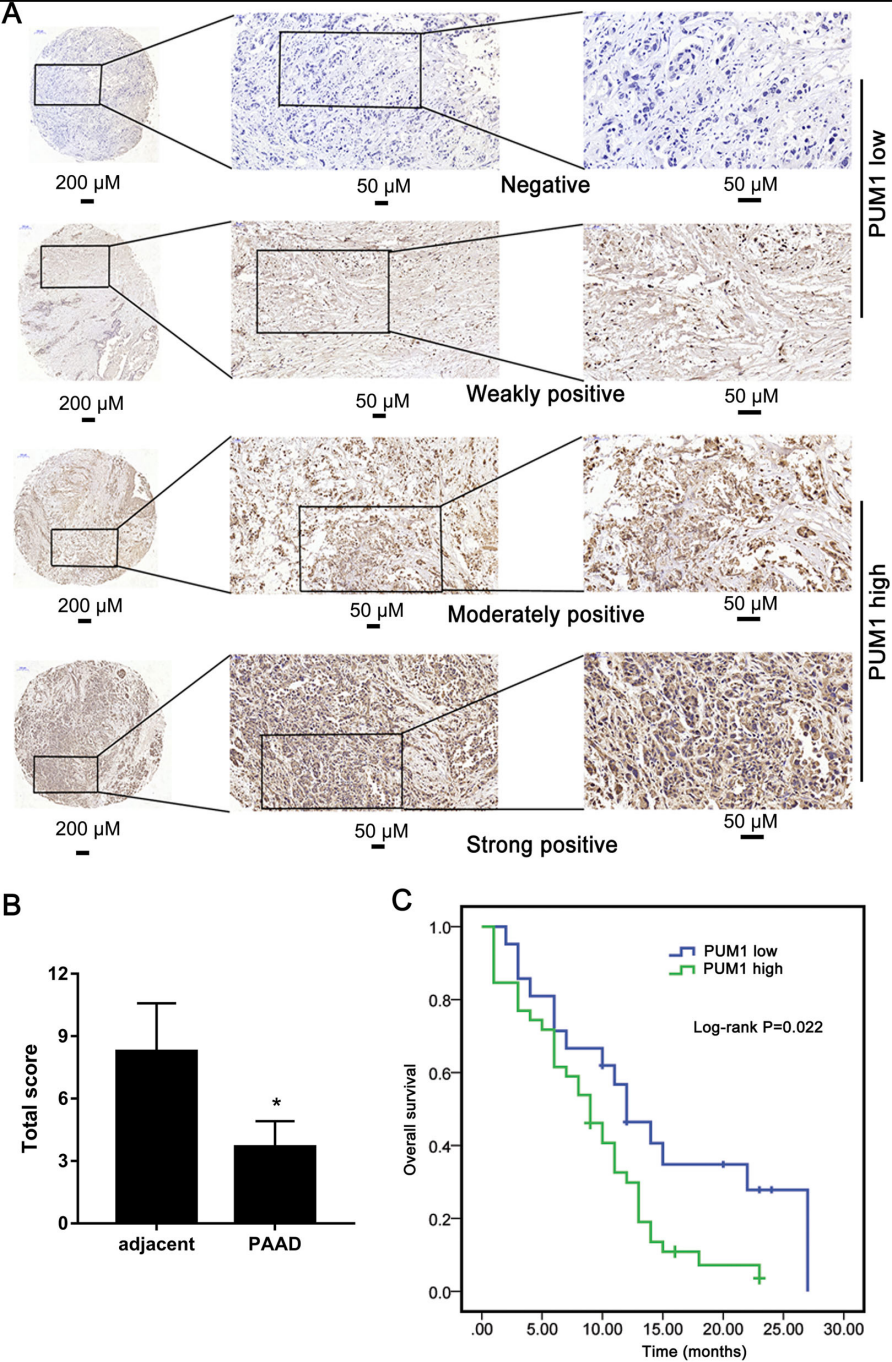
High PUM1 protein level indicated poor prognosis of pancreatic ductal adenocarcinoma (PDAC) patients. **a** Representative images of PUM1 expression by immunohistochemistry (IHC) analysis in tissue microarrays representing 60 PDAC patients. **b** Comparison of total scores for PUM1 assessment on tissue microarrays slides. Total score = positive staining scores × staining intensity score. *P < 0.05. **c** The overall survival rate of patients with PDAC was evaluated based on PUM1 expression using Kaplan–Meier analysis (*n* = 60, *P* = 0.022)

Analysis of the expression profiles of PUM1 mRNA in GEPIA revealed that PUM1 mRNA expression was higher in PAAD tissues than in adjacent tissues (Fig. 1a). We next analyzed the relationship between PUM1 mRNA expression and overall survival. The PUM1 mRNA expression levels were significantly associated with overall survival time, and low PUM1 mRNA level indicated poor prognosis (Fig. 1b). Further, we analyzed PUM1 protein levels in PDAC and adjacent tissues using tissue array analysis (Fig. 2a). PUM1 protein levels were higher in PDAC tissues than in adjacent tissues (Fig. 2b). We next analyzed the relationship between PUM1 protein levels and clinic characteristics, including age, sex, TNM stages, and overall survival time. PUM1 protein levels were not significantly associated with age and sex but were significantly associated with TNM stage (Table 1). Furthermore, PUM1 expression level was significantly associated with overall survival time, and high PUM1 protein level indicated poor prognosis (Fig. 2c).

**Table 1:** The relationship between PUM1 protein levels and clinic characteristics of patients with pancreatic adenocarcinoma

Characteristic	PUM1 level		*p*-value
	High expression *n* = 39	Low expression *n* = 21	
Age	56.32 ± 1232	59.24 ± 11.48	0.876
Sex			0.548
Male	21	13	
Female	18	8	
Ajcc_T staging			0.011
T1 + T2	29 (74.36)	20 (100)	
T3 + T4	10 (25.64)	0 (0)	
Ajcc_N staging (%)			0.034
N0	23 (58.97)	18 (85.71)	
N1 + N2	16 (41.03)	3 (14.29)	
Ajcc_M staging (%)			0.020
M0	27 (69.23)	20 (95.24)	
M1	12 (30.77)	1 (4.76)	
Ajcc_TNM_stage (%)			0.020
Stage I + II	27 (69.23)	20 (95.24)	
Stage III + IV	12 (30.77)	1 (4.76)	

### PUM1 knockdown inhibited cell proliferation and promoted apoptosis in vitro

**Fig. 3:**
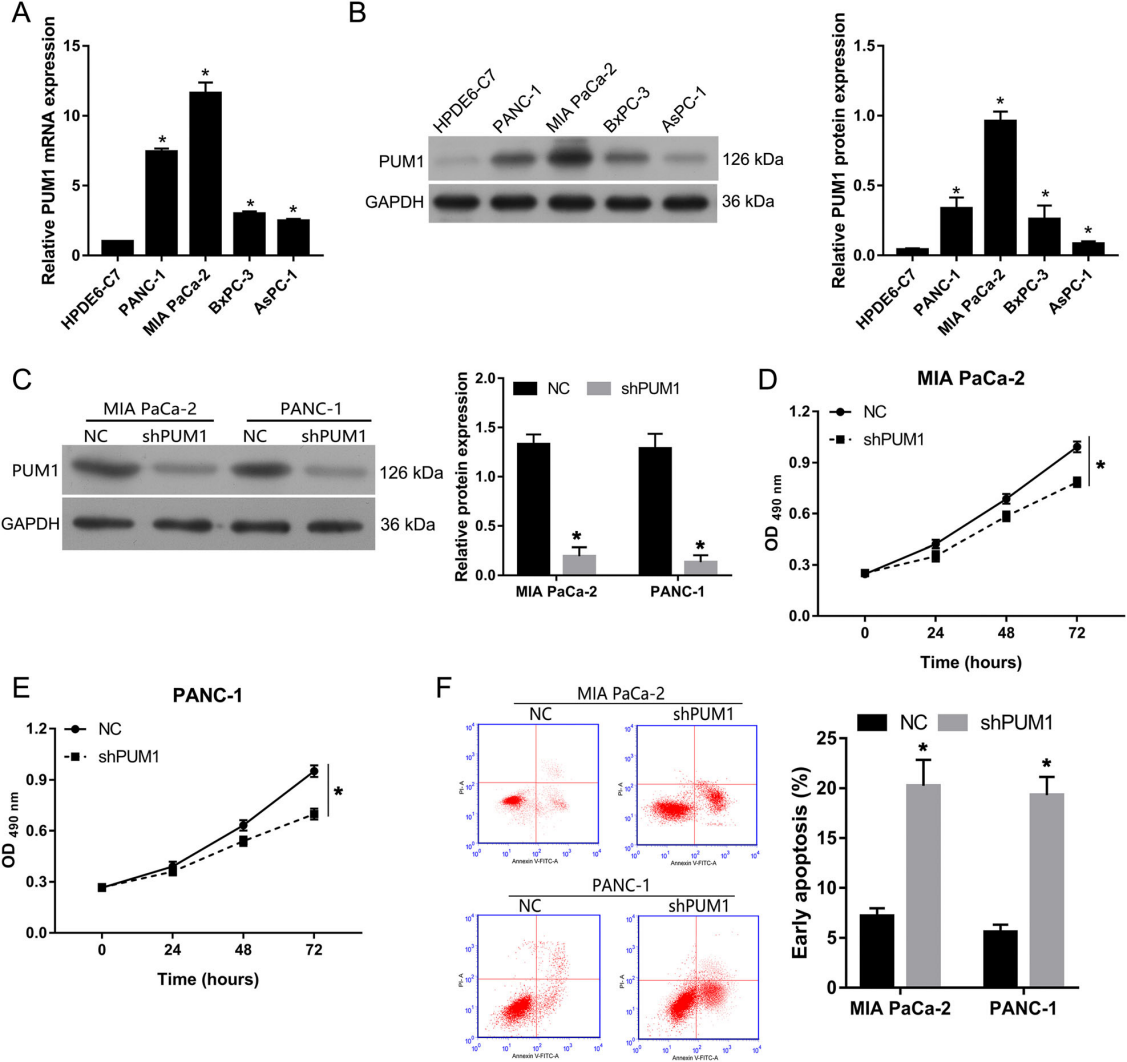
PUM1 knockdown inhibited cell proliferation and promoted apoptosis in MIA PaCa-2 and PANC-1 cells. PUM1 mRNA (**a**) and protein (**b**) expression levels in PDAC cell lines (BxPC-3, AsPC-1, MIA PaCa-2, and PANC-1) and human pancreatic duct epithelial cells HPDE6-C7. **c** PUM1 expression levels in cells infected with lentivirus expressing the shRNA of PUM1 (shPUM1) or negative (NC) control empty lentivirus. **d**, **e** Effect of PUM1 knockdown on the OD values at 490 nm. **f** Effect of PUM1 knockdown on early apoptosis rate. **b**, **c**, **f** The representative graphs are on the left, and the statistical results are on the right. *n* = 3, *P < 0.05, for **a**–**f**

As shown in Fig. 3a, b, PUM1 mRNA and protein expression levels were higher in PDAC cell lines (BxPC-3, AsPC-1, MIA PaCa-2, and PANC-1) than that in human pancreatic duct epithelial cells HPDE6-C7. Among the four PDAC cell lines, MIA PaCa-2, and PANC-1 cells had the highest PUM1 levels.

To investigate the role of PUM1 in PDAC cells, PUM1 knockdown MIA PaCa-2 and PANC-1 cells were established by transfecting them with lentivirus-expressing shPUM1. PUM1 levels were analyzed by western blot (Fig. 3c). Figure 3d, e shows that PUM1 knockdown decreased the OD value at 490 nm of MIA PaCa-2 and PANC-1 cells, indicating that PUM1 knockdown inhibited the proliferation of PDAC cells in vitro. In addition, we found that PUM1 knockdown increased the percentage of early apoptotic MIA PaCa-2 and PANC-1 cells, indicating that PUM1 knockdown promoted apoptosis in PDAC cells in vitro (Fig. 3f). Moreover, we also found that PUM1 overexpression promoted cell proliferation and suppressed apoptosis in MIA PaCa-2 cells (Fig. S1).

### PUM1 knockdown inhibited migration, invasion, and epithelial–mesenchymal transition (EMT) in vitro

**Fig. 4:**
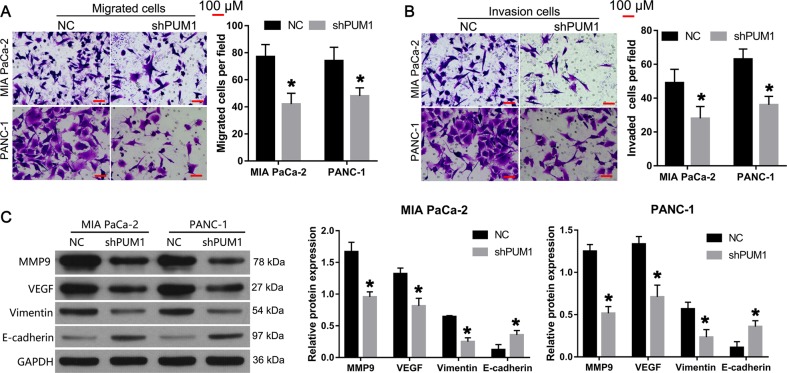
PUM1 knockdown inhibited migration, invasion, and EMT in MIA PaCa-2 and PANC-1 cells. Effect of PUM1 knockdown on cell migration (**a**) and invasion (**b**) detected by Transwell assays. Representative graphs (left). Bar graph showing the statistical result of migratory or invasive cells (right). **c** Effect of PUM1 knockdown on the expression of MMP9, VEGF, vimentin, and E-cadherin. Representative graphs of western blotting (left). Relative protein expression in reference to GAPDH (right). *n* = 3, *P < 0.05, for **a**–**c**

The results of the Transwell assays showed that the number of migratory and invasive MIA PaCa-2 and PANC-1 cells in the shPUM1 group was lower than that in the NC group (Fig. 4a, b). In addition, the expression levels of MMP9, VEGF, and vimentin decreased in the shPUM1 group compared with the NC group, while E-cadherin level increased (Fig. 4c). We also found that PUM1 overexpression promoted cell migration and invasion, increased the expression level of MMP9, VEGF, and vimentin, and decreased the expression level of E-cadherin in MIA PaCa-2 cells (Fig. S1).

### PUM1 knockdown inhibited tumor growth and metastasis in vivo

**Fig. 5:**
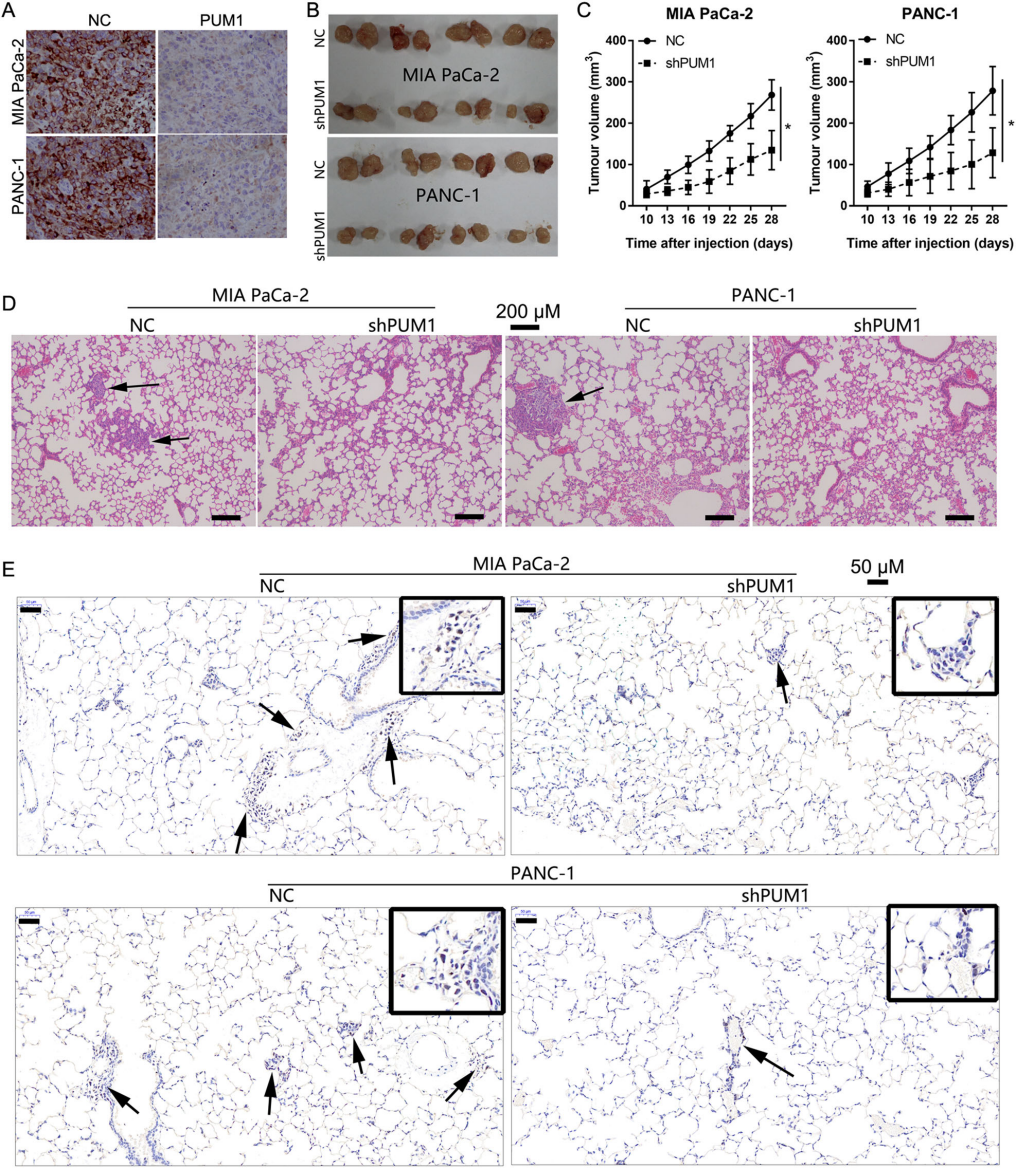
PUM1 knockdown inhibited cell proliferation, migration, and invasion in vivo. **a** PUM1 expression levels in tumors formed by the shPUM1 or NC group cells. **b**, **c** Effect of PUM1 knockdown on the growth of subcutaneous xenograft tumor. **b** Pictures of subcutaneous xenograft tumor. **c** Tumor growth curve. *n* = 8, *P < 0.05. **d**, **e** Effect of PUM1 knockdown on lung metastases examined by hematoxylin and eosin staining, or Ki67 expression detected by immunohistochemistry in the lung tissues of a lung metastasis mouse model

To further confirm the role of PUM1 in PDAC cells, we established a subcutaneous xenograft tumor mouse model and a lung metastasis mouse model. As shown in Fig. 5a, b, PUM1 expression levels were lower in the tumors formed by the shPUM1 group cells than in those formed by the NC group cells, and the size of tumors formed by the shPUM1 group cells was smaller than that of tumors formed by the NC group cells. The tumor growth curve also revealed that PUM1 knockdown inhibited the growth of subcutaneous tumors (Fig. 5c).

Furthermore, we found that mice in the shPUM1 group (MIA PaCa-2: six out of ten; PANC-1: six out of ten) exhibited less lung metastases compared with those in the NC group (MIA PaCa-2: eight out of ten; PANC-1: nine out of ten). The H&E staining showed that the lung tissues of mice injected with shPUM1 group cells had less metastatic foci than those injected with NC group cells (Fig. 5d). In addition, less Ki67-positive cells were found in the lung tissues of mice injected with shPUM1 group cells when compared with mice injected with NC group cells (Fig. 5e).

### PUM1 knockdown activated the PERK/eIF2/ATF4 signaling pathway

**Fig. 6:**
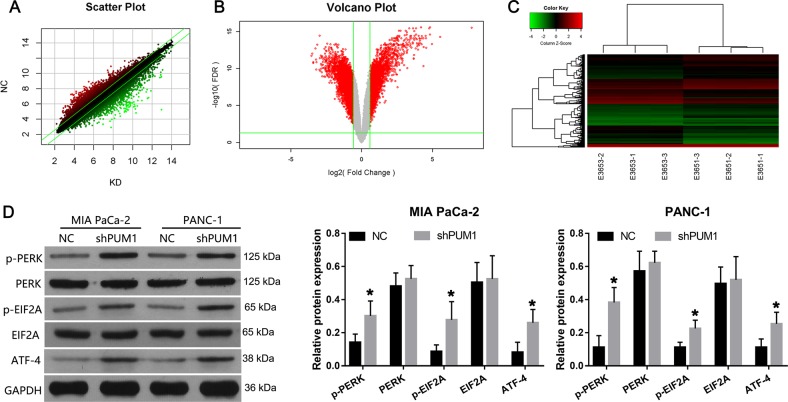
PUM1 knockdown activated the PERK/eIF2/ATF4 signaling pathway. Volcano plot (**a**), scatter plot (**b**), and cluster gram (**c**) of the cDNA microarrays. **d** Effect of PUM1 knockdown on the expression of key components of the eIF2 signaling pathway. Left: Representative graphs of western blotting. Right: Statistical result of the relative protein expression in reference to GAPDH. *n* = 3, *P < 0.05

To further explore the mechanisms of PUM1 in PDAC cells, the mRNA expression profile in PUM1 knockdown was examined using cDNA microarrays (Fig. 6a–c). As shown in Table S1, 1768 upregulated and 2864 downregulated mRNAs were identified in the PUM1 knockdown MIA PaCa-2 cells when compared with the NC MIA PaCa-2 cells. Through IPA analysis, we found that the eIF2 signaling pathway has significant correlations with PUM1 knockdown (Fig. S2). Next, the expression of key components of the eIF2 signaling pathway was verified by western blotting in both PUM1 knockdown MIA PaCa-2 and PANC-1 cells. The results showed that the levels of p-PERK, p-EIF2A, and ATF4 were higher in the PUM1 knockdown MIA PaCa-2 and PANC-1 cells compared with the NC MIA PaCa-2 and PANC-1 cells, indicating that PUM1 knockdown activated the PERK/eIF2/ATF4 signaling pathway (Fig. 6d). Moreover, we found that PUM1 overexpression decreased the levels of p-PERK, p-EIF2A, and ATF4 in MIA PaCa-2 cells, indicating that PUM1 overexpression inactivated the PERK/eIF2/ATF4 signaling pathway (Fig. S1).

### PUM1 level has a significant negative correlation with p-PERK level

**Fig. 7:**
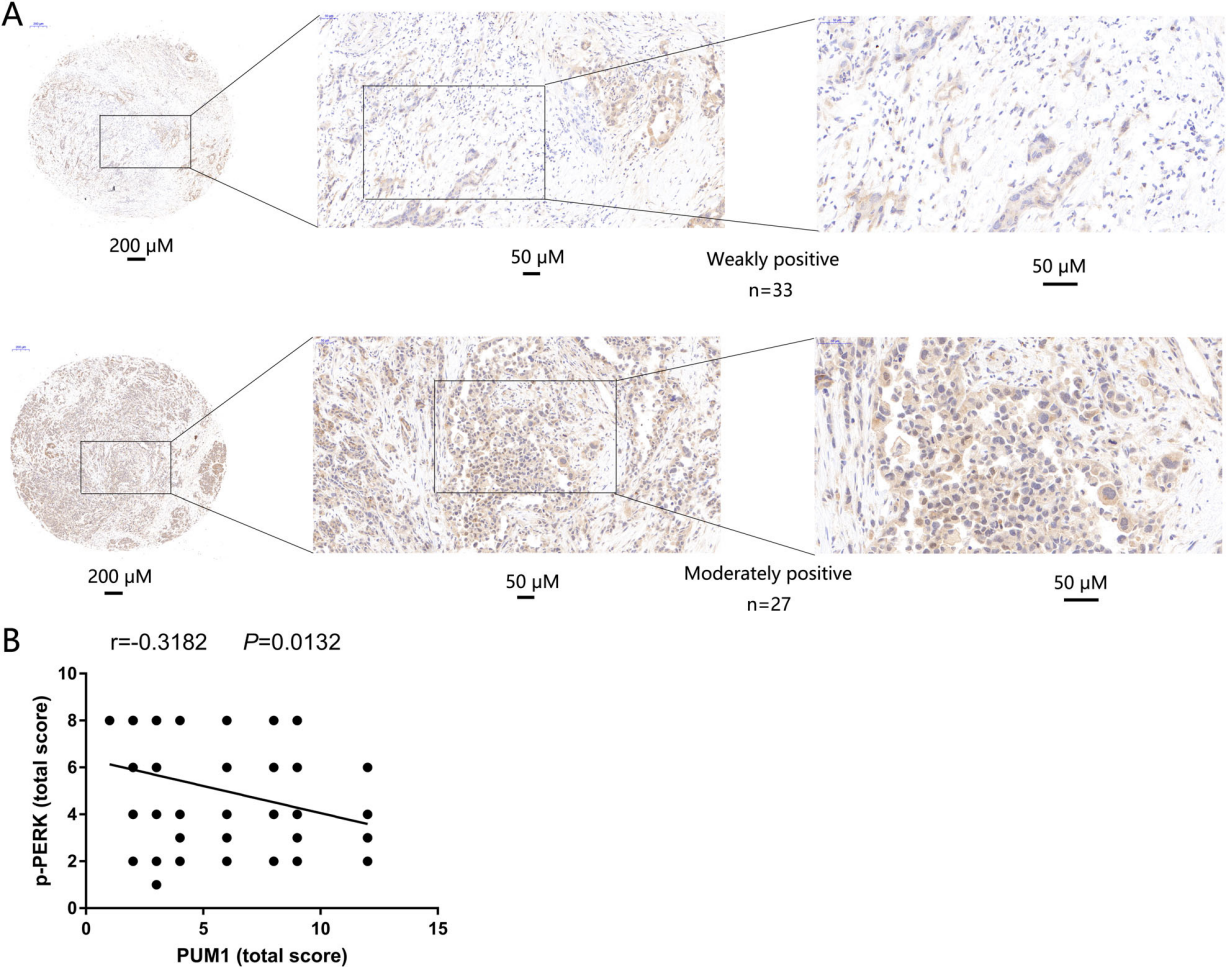
PUM1 levels had a significant negative correlation with p-PERK levels. **a** Representative pictures showing p-PERK levels in 60 PDAC tissues on tissue microarray examined by immunohistochemical analysis. **b** Linear regression analysis showing the correlation of PUM1 and p-PERK levels in PDAC tissues (*n* = 60)

To further confirm the relationship between PUM1 and the PERK/eIF2/ATF4 signaling pathway, we analyzed the correlation between PUM1 and p-PERK levels in PDAC tissues. As shown in Fig. 7a, among 60 PDAC tissues, p-PERK levels were weakly positive in 33 and moderately positive in 27. The results of the linear regression analysis showed that PUM1 levels had a significant negative correlation with p-PERK levels (*r* = −0.3182, *P* *=* 0.0132) (Fig. 7b).

### PERK overexpression inhibited cell proliferation, migration, invasion, and EMT, and promoted apoptosis in vitro

**Fig. 8:**
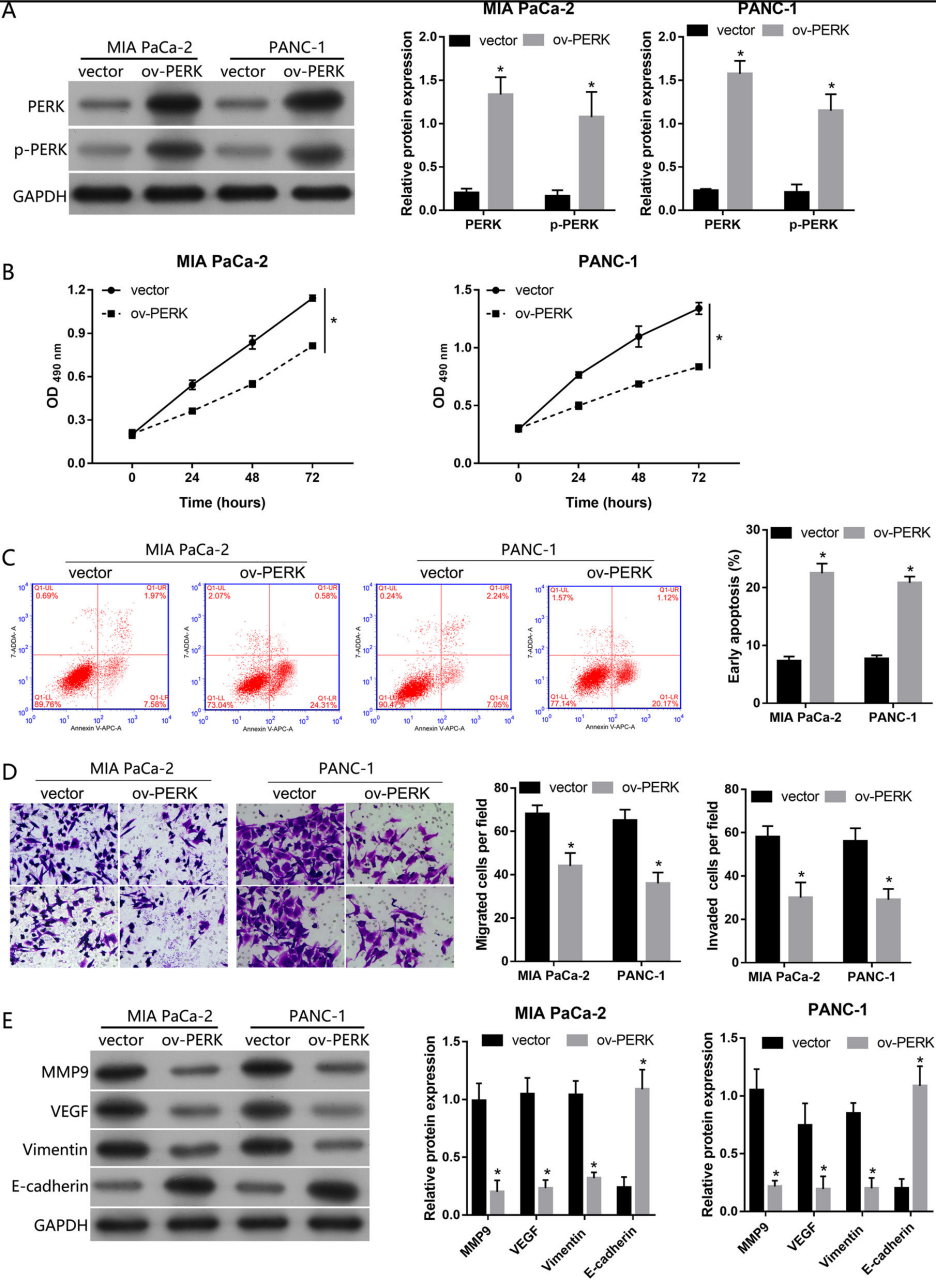
PERK overexpression inhibited cell proliferation, migration, invasion, and EMT and promoted apoptosis in MIA PaCa-2 and PANC-1 cells. **a** PERK expression levels in MIA PaCa-2 and PANC-1 cells transfected with PERK overexpression vector (ov-PERK) or empty vector (vector). **b** Effect of PERK overexpression on the OD value at 490 nm. **c** Effect of PERK overexpression on early apoptosis rate. **d** Effect of PERK overexpression on cell migration and invasion detected by Transwell assays. **e** Effect of PERK overexpression on the expression of MMP9, VEGF, vimentin, and E-cadherin. **a**, **c**–**e** The representative graphs are on the left, and the statistical results are on the right. *n* = 3, *P < 0.05

To investigate the role of PERK in PDAC cells, PERK-overexpressing MIA PaCa-2 and PANC-1 cells were established by transfecting them with plasmids expressing PERK, and PERK levels were analyzed by western blot (Fig. 8a).

p-PERK level was increased in ov-PERK transfected cells when compared with vector-transfected cells (Fig. 8a). PERK overexpression decreased the OD value at 490 nm in both MIA PaCa-2 and PANC-1 cells (Fig. 8b), indicating that PERK overexpression inhibited the proliferation of PDAC cells in vitro. In addition, we found that PERK overexpression increased the percentage of early apoptotic MIA PaCa-2 and PANC-1 cells, indicating that PERK overexpression promoted the apoptosis of PDAC cells in vitro (Fig. 8c). The results of the Transwell assays showed that the number of migratory and invasive MIA PaCa-2 and PANC-1 cells in the ov-PERK group was lower than that in the vector group (Fig. 8d). In addition, the expression levels of MMP9, VEGF, and vimentin decreased in the ov-PERK group compared with the vector group, while the E-cadherin level increased (Fig. 8e).

### PERK inhibitor alleviated the effect of PUM1 knockdown on cell proliferation and apoptosis in vitro

**Fig. 9:**
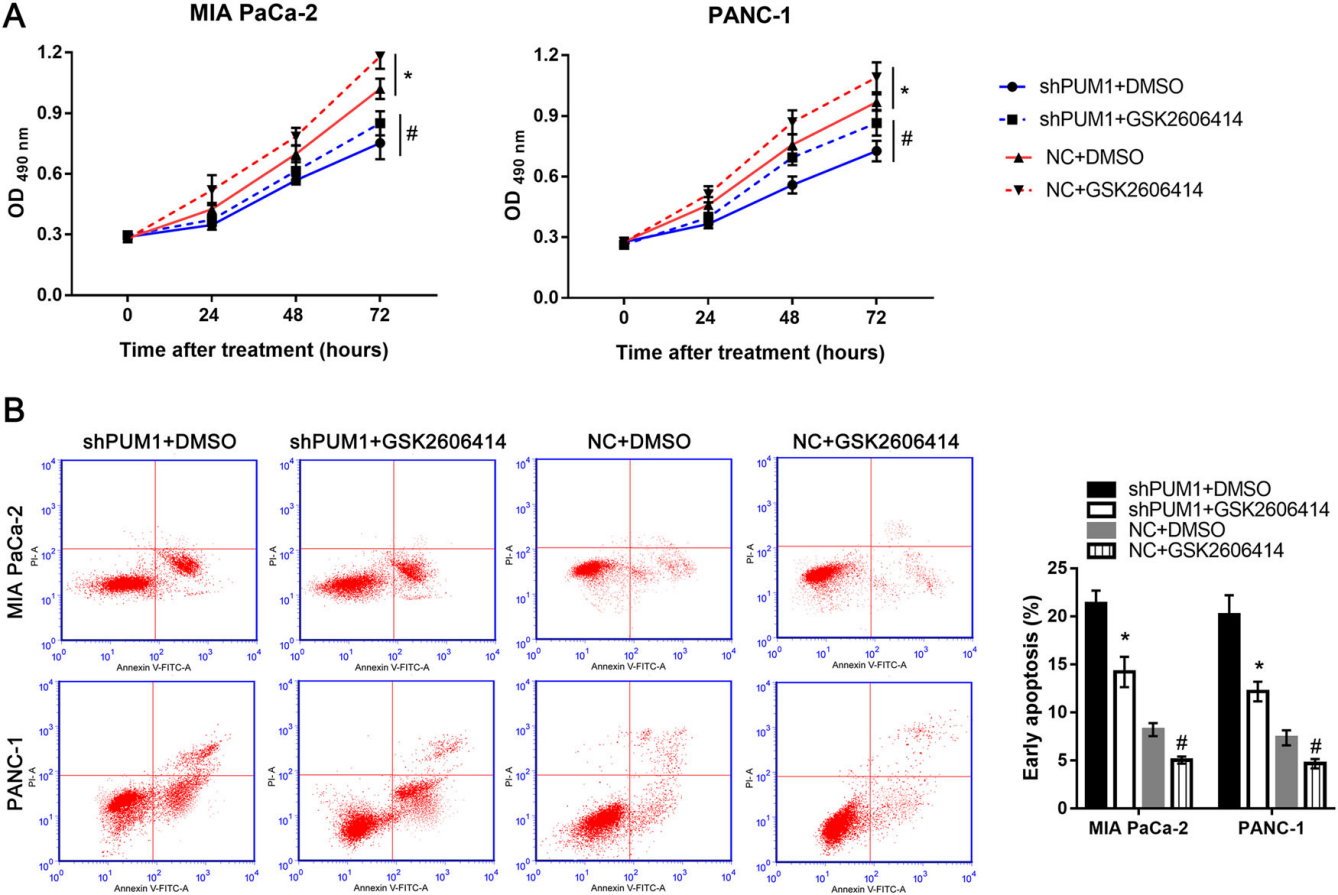
PERK inhibitor GSK2606414 alleviated the effects of PUM1 knockdown on cell proliferation and apoptosis in MIA PaCa-2 and PANC-1 cells. **a** Effect of the PERK inhibitor GSK2606414 or DMSO on OD value at 490 nm in shRNA or NC cells. **b** Effect of PERK inhibitor GSK2606414 or DMSO on early apoptosis rate in shRNA or NC cells. Left: Representative graphs of flow cytometry analysis for apoptosis. Right: Statistical result of early apoptosis rate for each group. *n* = 3, *P < 0.05, shPUM1 + DMSO vs. shPUM1 + GSK2606414; *n* = 3, ^#^*P* < 0.05, NC + DMSO vs. NC + GSK2606414

To further confirm the role of the PERK/eIF2/ATF4 signaling pathway in PUM1 knockdown-induced suppression of proliferation and promotion of apoptosis, the PERK inhibitor, GSK2606414, was used to weaken the activation induced by PUM1 knockdown. The OD value at 490 nm in the shPUM1 + GSK2606414 group was higher than that in shPUM1 + DMSO group in both MIA PaCa-2 and PANC-1 cells, but lower than that in the NC + DMSO group (Fig. 9a). Additionally, the percentage of early apoptosis in the shPUM1 + GSK2606414 group was less than that in the shPUM1 + DMSO group in both MIA PaCa-2 and PANC-1 cells, but more than that in the NC + DMSO group (Fig. 9b). These results indicated that the PERK inhibitor alleviated the effect of PUM1 knockdown on cell proliferation and apoptosis. However, this reversal effect induced by GSK2606414 was partial. Moreover, we found that the OD value at 490 nm in the NC + GSK2606414 group was higher than that in NC + DMSO group, and the percentage of early apoptosis in the NC + GSK2606414 group was less than that in NC + DMSO group in both MIA PaCa-2 and PANC-1 cells (Fig. 9a, b).

### PERK inhibitor alleviated the effect of PUM1 knockdown on cell migration, invasion, and EMT in vitro

**Fig. 10:**
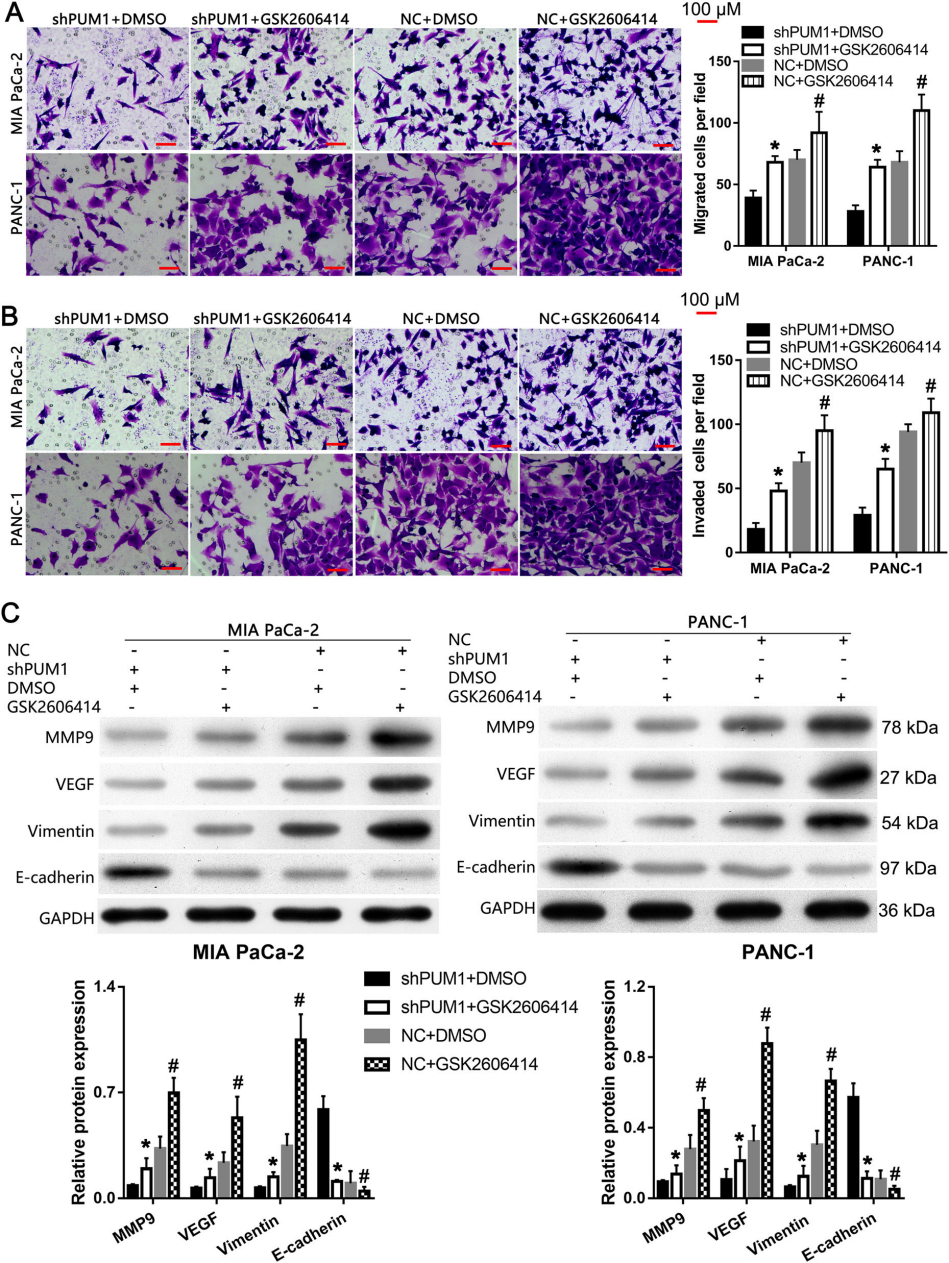
PERK inhibitor alleviated the effect of PUM1 knockdown on cell migration, invasion, and EMT in MIA PaCa-2 and PANC-1 cells. Effect of the PERK inhibitor GSK2606414 or DMSO on cell migration (**a**) and invasion (**b**) in shRNA or NC cells detected by Transwell assays. Left: Representative graph. Right: Bar graph of the statistical result of the migratory or invasive cells. **c** Effect of the PERK inhibitor GSK2606414 or DMSO on the expression of MMP9, VEGF, vimentin, and E-cadherin in shRNA or NC cells. Up: Representative graph of western blotting. Bottom: Statistical result of the relative protein expression in reference to GAPDH. *n* = 3, *P < 0.05, shPUM1 + DMSO vs. shPUM1 + GSK2606414; *n* = 3, ^#^*P* < 0.05, NC + DMSO vs. NC + GSK2606414, for **a**–**c**

The results of the Transwell assays showed that the number of migratory and invasive MIA PaCa-2 and PANC-1 cells in the shPUM1 + GSK2606414 group was higher than that in shPUM1 + DMSO group (Fig. 10a, b). In addition, we found that the expression levels of MMP9, VEGF, and vimentin were increased in the shPUM1 + GSK2606414 group when compared with the shPUM1 + DMSO group, whereas the E-cadherin levels were increased (Fig. 10c). Moreover, we found that the number of migratory and invasive MIA PaCa-2 and PANC-1 cells was greater, the expression levels of MMP9, VEGF, and vimentin were higher, and the E-cadherin level was lower in the NC + GSK2606414 group than in the NC + DMSO group (Fig. 10).

## Discussion

PUM1, also known as HSPUM, PUMH1, PUML1, or SCA47, contains a sequence-specific RNA-binding domain and can bind to the 3′ untranslated regions of mRNAs such as siah E3 ubiquitin protein ligase 1and E2F transcription factor 3^5,15^. Recently, numerous studies revealed that PUM1 is an important protein in many physiological processes^16,17^. In humans, its role has been reported in ovarian cancer and NSCLC^6,7^. However, the function of PUM1 in PDAC had not been reported. Through bioinformatics analysis of GEPIA and TCGA databases, we found that PUM1 mRNA was higher in PAAD tissues than in adjacent tissues and decreased PUM1 mRNA expression levels were associated with poor prognosis of patients with PAAD. Therefore, it is necessary to further evaluate the role of PUM1 in PDAC.

The tissue array analysis showed that PUM1 protein levels were higher in PDAC tissues than in adjacent tissues. In addition, the PUM1 expression level was higher in PDAC cell lines than that in human pancreatic duct epithelial cells. Our results are consistent with the bioinformatics analysis of the PUM1 mRNA expression level in PAAD tissues, and with a previous study that reported that PUM1 protein levels were higher in ovarian cancer tissues than in normal tissues^6^. Subsequently, we found that the increased PUM1 level significantly associated with the TNM stage, which is consistent with the report on ovarian cancer^6^.

Moreover, we evaluated the relationship between PUM1 and prognosis. Although we found that PUM1 mRNA and protein expression levels were associated with overall survival time, our conclusions are inconsistent. Our retrospective study showed that increased PUM1 protein levels indicated a poor prognosis, but the analysis from TCGA revealed that a low PUM1 mRNA level indicated poor prognosis. This contradiction may be due to two reasons: (1) The results from TCGA data are based on the PUM1 mRNA level analyzed by mRNA-sequencing or mRNA Chip, while our results are based on the PUM1 protein level analyzed by immunohistochemical analysis using tissue microarrays. (2) Most patients enrolled in the TCGA project were Caucasian, while most patients enrolled in our study were Asian. Because PUM1 is a functional protein, we believe that the result evaluating PUM1 protein expression and its association with prognosis is more valuable and credible for clinical use and as a literature resource.

The in vitro functional analysis demonstrated that PUM1 knockdown inhibited cell proliferation, migration, and invasion, and promoted apoptosis in MIA PaCa-2 and PANC-1 cells; this result is consistent with the report on ovarian cancer^6^. In the present study, we also performed a functional analysis in vivo to further evaluate the role of PUM1 in PDAC. Our results showed that PUM1 knockdown can inhibit the growth of subcutaneous xenograft tumor and decrease the number of metastatic foci and Ki67-positive cells in the lung tissues of a lung metastasis mice model. Ki67 is highly expressed in malignant cells and is a marker of cancer proliferation^18^. Thus, the in vivo results of the present study support that PUM1 knockdown inhibited tumor growth and metastasis in vivo.

Moreover, we found that PUM1 overexpression promoted cell proliferation, migration, and invasion, and suppressed apoptosis in MIA PaCa-2 cells. All these results demonstrated that PUM1 functioned as an oncogene by promoting cell proliferation, migration, and invasion in PDAC. Hence, PUM1 is a potential therapeutic target for the treatment of this type of cancer.

We also found that PUM1 knockdown decreased the expression levels of vimentin and increased the expression levels of E-cadherin. Vimentin is a mesenchymal marker, and E-cadherin is an epithelial marker in the process of EMT^19^. Our results indicated that PUM1 knockdown suppressed EMT in PDAC. To the best of our knowledge, this is the first report demonstrating the involvement of PUM1 in EMT regulation. The activation of EMT allows cancer cells to acquire migratory and invasive properties during tumorigenesis^20^. In the present study, we found that PUM1 knockdown not only inhibited migration and invasion but also suppressed EMT. Moreover, PUM1 overexpression promoted migration, invasion, and EMT in MIA PaCa-2. Therefore, we predicted that PUM1 may be involved in promoting migration and invasion by activating EMT in PDAC. However, this prediction needs to be further confirmed in future studies.

Through cDNA microarrays and IPA analysis, we found that the activation of the eIF2 signaling pathway has a significant correlation with PUM1 knockdown. These results were further confirmed by the increased protein levels of key components of the eIF2 signaling pathway in PUM1 knockdown cells. Moreover, PERK inhibitor GSK2606414 alleviated the effect of PUM1 knockdown on cell proliferation, apoptosis, cell migration, invasion, and EMT in vitro. These results demonstrated that PUM1 functioned as an oncogene in PDAC by suppressing PERK/eIF2/ATF4 signaling pathway.

Numerous studies have revealed that unfolded protein response (UPR), a pathway triggered by ERS, regulates multiple pathological processes in cancer cells^21–23^. UPR is a double-edged sword for cancer development, as^24^ it suppresses tumor cell proliferation but also induces tumor cell survival, progression, and metastasis under unfavorable conditions^24^.

As a key regulator of UPR^8^, the PERK/eIF2/ATF4 signaling pathway also regulates tumor progression^25^. However, most studies addressing the role of the PERK/eIF2/ATF4 signaling pathway in tumor cells focus on its role during UPR. It has been reported that PERK/eIF2alpha/ATF4 activation during UPR increases tolerance to extreme hypoxia and promotes tumor growth under hypoxia condition^26,27^. In addition, PERK/eIF2alpha/ATF4 activation also can potentiate apoptosis in cancer cells^28,29^.

No study had previously addressed the role of the PERK/eIF2/ATF4 signaling pathway in PDAC. Here, we demonstrated that PUM1 functioned as an oncogene in PDAC by suppressing the PERK/eIF2/ATF4 signaling pathway. In addition, to the best of our knowledge, the present study is the first to determine that PERK overexpression inhibited cell proliferation, migration, invasion, and EMT, and promoted apoptosis in PDAC cells. Our results are not consistent with the role of PERK in UPR because all the cells in our study were cultured under favorable conditions. Therefore, we predicted that the PERK/eIF2/ATF4 signaling pathway may play a different role in cells cultured in favorable and unfavorable conditions. In addition, the PERK/eIF2/ATF4 signaling pathway may be involved in other pathways except UPR activation. Therefore, further studies should illuminate the role of the PERK/eIF2/ATF4 signaling pathway in tumor progression.

Our study has some limitation. First, other signaling pathways were also identified in cDNA microarrays and IPA analysis. Further studies are needed to better elucidate the regulatory mechanism of PUM1 in apoptotic, proliferative, invasive, and metastatic behaviors in PDAC cells. Second, the mechanism by which PUM1 modulates the PERK/eIF2/ATF4 pathway remains unknown and should be addressed in future studies.

In conclusion, we found that PUM1 protein levels are higher in PDAC tissues than in adjacent tissues, and PDAC patients with high PUM1 protein expression tended to have a relatively more advanced TNM stage and short survival time. Thus, high PUM1 protein expression may be an independent prognostic factor in patients with PDAC. In addition, PUM1 knockdown inhibited cell proliferation, migration, invasion, and EMT, and promoted apoptosis in vitro and in vivo, indicating that PUM1 functioned as an oncogene in PDAC. To our knowledge, this is the first study to reveal that PUM1 is related to the development and prognosis of PDAC.

Moreover, we elucidated the relevant molecular mechanism of PUM1 in PDAC. PUM1 knockdown suppressed cell growth, invasion, and metastasis and promoted apoptosis by activating the PERK/eIF2/ATF4 signaling pathway in PDAC cells. Our study may provide a potential target for pharmaceutical development and novel therapeutic strategies for the use of PUM1 inhibitors or activators of the PERK/eIF2/ATF4 signaling pathway to control PDAC invasion and metastasis in the future.

## Supplementary information

supplementary_figures

Table S1
