# Supplementary material for: PUM1 knockdown prevents tumor progression by activating the PERK/eIF2/ATF4 signaling pathway in pancreatic adenocarcinoma cells
Source: Cell Death Dis. 2019 Aug 8;10(8):595. doi: 10.1038/s41419-019-1839-z (PMC6687830; doi:10.1038/s41419-019-1839-z)
Supplement: Supplementary file 1 — supplementary_figures [file 41419_2019_1839_MOESM1_ESM.docx]

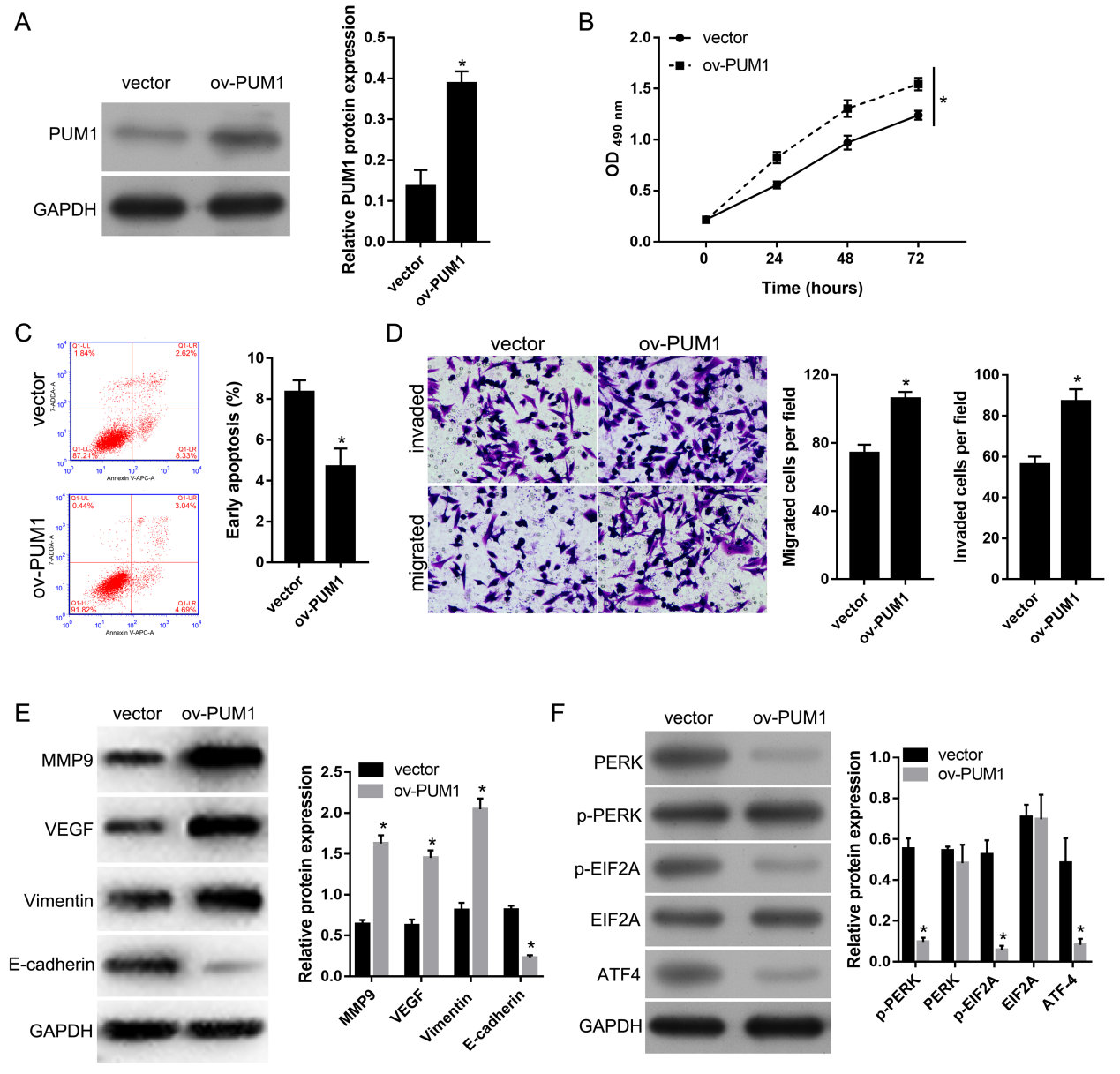


**Figure S1**. PUM1 overexpression promoted cell proliferation, migration, invasion, and EMT and inhibited apoptosis in MIA PaCa-2 cells. A: PUM1 expression levels in MIA PaCa-2 cells transfected with PUM1 overexpression vector (ov-PUM1) or empty vector (vector). B: Effect of PUM1 overexpression on OD value at 490 nm. C: Effect of PUM1 overexpression on early apoptosis rate. D: Effect of PUM1 overexpression on cell migration and invasion detected by Transwell assays. E: Effect of PUM1 overexpression on the expression of MMP9, VEGF, Vimentin, and E-cadherin. F: Effect of PUM1 overexpression on the expression of key components of the eIF2 signaling pathway. For panels A, C, D, E and F, the representative graphs are on the left, and the statistical results are on the right. * P < 0.05, for all panels.


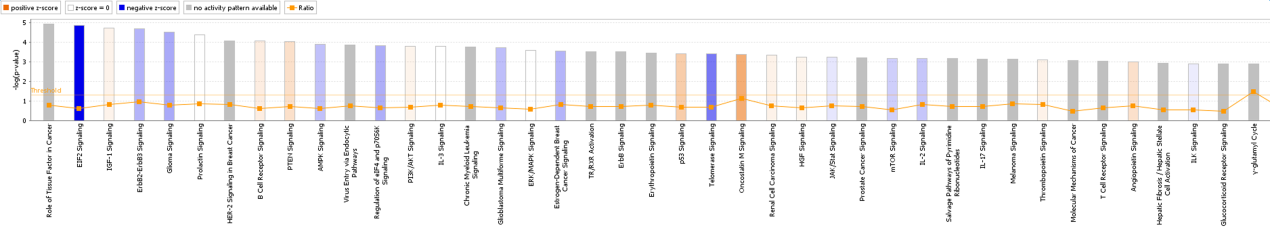


Figure S2 Results of the Ingenuity Pathway Analysis (IPA).
